# Are investigators’ access to trial data and rights to publish restricted and are potential trial participants informed about this? A comparison of trial protocols and informed consent materials

**DOI:** 10.1186/s12910-021-00681-9

**Published:** 2021-08-28

**Authors:** Asger S. Paludan-Müller, Michelle C. Ogden, Mikkel Marquardsen, Karsten J. Jørgensen, Peter C. Gøtzsche

**Affiliations:** 1 Center for Evidence-Based Medicine Odense and Cochrane Denmark, Department of Clinical Research, University of Southern Denmark, JB Winsløwsvej 9b, 3rd floor, 5000 Odence, Denmark; 2 Open Patient data Exploratory Network (OPEN), Odense University Hospital, Odense, Denmark; 3 Nordic Cochrane Centre, Rigshospitalet, Copenhagen, Denmark; 4 Institute for Scientific Freedom, Copenhagen, Denmark

**Keywords:** Research ethics, Informed consent, Publication rights, Publication bias, Clinical study protocols

## Abstract

**Objectives:**

To determine to which degree industry partners in randomised clinical trials own the data and can constrain publication rights of academic investigators.

**Methods:**

Cohort study of trial protocols, publication agreements and other documents obtained through Freedom of Information requests, for a sample of 42 trials with industry involvement approved by ethics committees in Denmark. The main outcome measures used were: proportion of trials where data was owned by the industry partner, where the investigators right to publish were constrained and if this was mentioned in informed consent documents, and where the industry partner could review data while the trial was ongoing and stop the trial early.

**Results:**

The industry partner owned all data in 20 trials (48%) and in 16 trials (38%) it was unclear. Publication constraints were described for 30 trials (71%) and this was not communicated to trial participants in informed consent documents in any of the trials. In eight trials (19%) the industry partner could review data during the trial, for 20 trials (48%) it was unclear. The industry partner could stop the trial early without any specific reason in 23 trials (55%).

**Conclusions:**

Publication constraints are common, and data is often owned by industry partners. This is rarely communicated to trial participants. Such constraints might contribute to problems with selective outcome reporting. Patients should be fully informed about these aspects of trial conduct.

## Background

Cooperation between pharmaceutical companies and academic investigators is common for randomised clinical trials (RCTs) [1, 2]. While this has advantages, it is essentially a business transaction and conflicts of interest abound. There is convincing empirical evidence of selective reporting of results in industry funded trials [1, 3], and industry trials are less likely to be published than non-industry trials [4, 5].

The World Medical Association’s Declaration of Helsinki states that “researchers, authors, sponsors, editors and publishers all have ethical obligations with regard to the publication and dissemination of the results of research. Researchers have a duty to make publicly available the results of their research on human subjects” and that “Negative and inconclusive as well as positive results must be published or otherwise made publicly available.”[6]

However, it may be difficult for investigators in industry-sponsored trials to adhere to these requirements, as their rights to publish may be constrained. Previous studies have examined constraints on publication rights in industry-initiated trials. In 2006, a study found that 40 of 44 (91%) trials approved by ethics committees in Denmark between 1994 and 1995 described constraints on publication for participating clinicians in the trial protocol and the same was true for 41 of 44 trials (93%) approved in 2004 [7]. In 2016, a study examined whether there were constraints on publication in 647 protocols approved by ethics committees in Switzerland and Germany between 2000 and 2003. Four-hundred-fifty-six (70%) trial protocols mentioned publication agreements and in 393 of those (86%) the industry partner had the right to either disapprove or at the least review publications [8].

Both studies used relatively old samples. To our knowledge, no study has examined publication constraints in a recent sample of randomised clinical trials (RCTs) approved by ethics committees. Additionally, none of the previous studies have compared information on publication restraints available to ethics committees with the information provided to research participants, who should be informed about key conditions of the trial prior to making an informed decision according to the Helsinki Declaration. As altruism is generally considered an important reason for participating in clinical trials [9, 10], it is important that patients are informed of potential publication constraints.

Another potentially problematic issue in clinical trials is early stopping. A 2010 review found that for trials stopping prematurely for benefit, effects were exaggerated by 29% compared to trials of the same intervention that had not stopped early and this bias persisted regardless of whether stopping rules were pre-defined [11]. In the 2006 study, the industry sponsor had access to accumulating data in 16 out of 44 trials (36%) and the sponsor could stop the trial at any time, for any reason, in an additional 16 trials (36%) [7].

In this study we examined to which degree access to data and the right to publish is restricted, whether this is communicated to patients, and whether the industry partner has the opportunity to accumulate data and stop the trial prematurely. We used a sample of relatively recent RCTs approved by ethics committees in Denmark. This sample was also used to examine to which degree trial rationale and choice of comparator was justified through prior literature reviews [12] and whether potential trial participants were adequately informed of benefits and harms associated with participating in the trial.

## Methods

### Access to clinical trial protocols

As described elsewhere [12] we gained access to clinical study protocols and other documents submitted to Danish ethics committees through Freedom of Information requests.

We included protocols from parallel group RCTs with industry involvement from all clinical fields. We excluded trials with only surrogate primary outcomes, as it requires detailed content area expertise from diverse clinical fields to determine the clinical relevance of such outcomes.

### Identification and retrieval of trial documents

The Danish National Committee on Health Research Ethics collects information about all trials given ethical approval by regional ethics committees in Denmark and publishes this information on their website [13]. We used this website to identify all clinical trials approved between January 2012 and March 2013, and then we used information from the website to identify the trials in trial registries (clinicaltrials.gov, the EU Clinical Trials Register, and the WHO International Clinical Trial Registry Platform) [14–16]. We used this information to identify potentially eligible trials but limited the period of inclusion to October 1 2012 to March 31 2013, as we identified substantially more trials than we could include in our analysis.

For eligible trials, we submitted Freedom of Information requests to the regional ethics committees in Denmark to obtain the following documents: Clinical study protocols, informed consent documents, publication agreements between study sponsors and investigators, financial agreements between study sponsors and investigators, and any other relevant documents. We used the protocols to make a final assessment of eligibility.

The process of identifying and retrieving relevant trial documents is described in detail elsewhere [12].

### Data extraction

All data were extracted by one researcher and checked by another researcher. Any discrepancies were solved through discussion, potentially involving a third researcher.

#### Characteristics of included trials

For all trials, we extracted the following characteristics from the protocols: medical specialty, experimental intervention and comparator(s), number of arms, single-site or multi-centre study, planned sample size, funding source, trial duration, primary outcomes, and trial phase.

We determined whether trials were partially or fully industry sponsored. We considered a trial fully industry sponsored when a commercial entity was the primary or only sponsor and partially industry sponsored when the primary sponsor was a non-commercial entity but a commercial entity provided either medication, devices, manpower or funding for the trial.

#### Information on rights to data and publication constraints

We extracted information on the roles and responsibilities of sponsors, ownership of data and rights to access data, as well as whether publication constraints existed and the nature of such constraints. The information was extracted from the protocols and other relevant documents (e.g. publication agreements or layperson summaries in Danish).

#### Information on sponsor’s ability to accumulate data during the trial and early stopping rules

We extracted information on the sponsor’s ability to review data while the study was ongoing, e.g. through interim analyses or through participation in data monitoring committees (DMCs), and information on the sponsor’s ability to stop the trial early, including pre-defined stopping rules.

### Analysis

The extracted information was assessed according to our six pre-specified questions.

1. Were the roles and responsibilities of the trial funders and sponsors described?
2. Who owned the data accumulated during the trial?
3. Were the investigators’ rights to publish restricted?

We particularly assessed whether there were restrictions to the time-period for which investigators could publish; if the sponsor had the right to review and comment on potential publications; if investigators were obliged to take the comments from sponsors into consideration; and whether sponsors could delay or prevent publication.

4. Was information about potential publication constraints described in the informed consent document?
5. Did the industry partner have the opportunity to review data during the study?
6. Could the industry partner stop the trial early?

If yes, we determined whether this could be done for any reason, or whether there were pre-defined stopping rules.

All assessments were checked by a second researcher. Disagreements were discussed with a senior researcher. In cases of doubt, we conservatively assumed that the reply was the one that would generally be considered most positive, e.g., for question 1 we assumed the sponsors role was described, for question 2 we assumed that the trialists owned the data, and for question 5 and 6 we assumed that the answer was no.

We present descriptive statistics for trial characteristics and for these assessments.

## Results

**Fig. 1:**
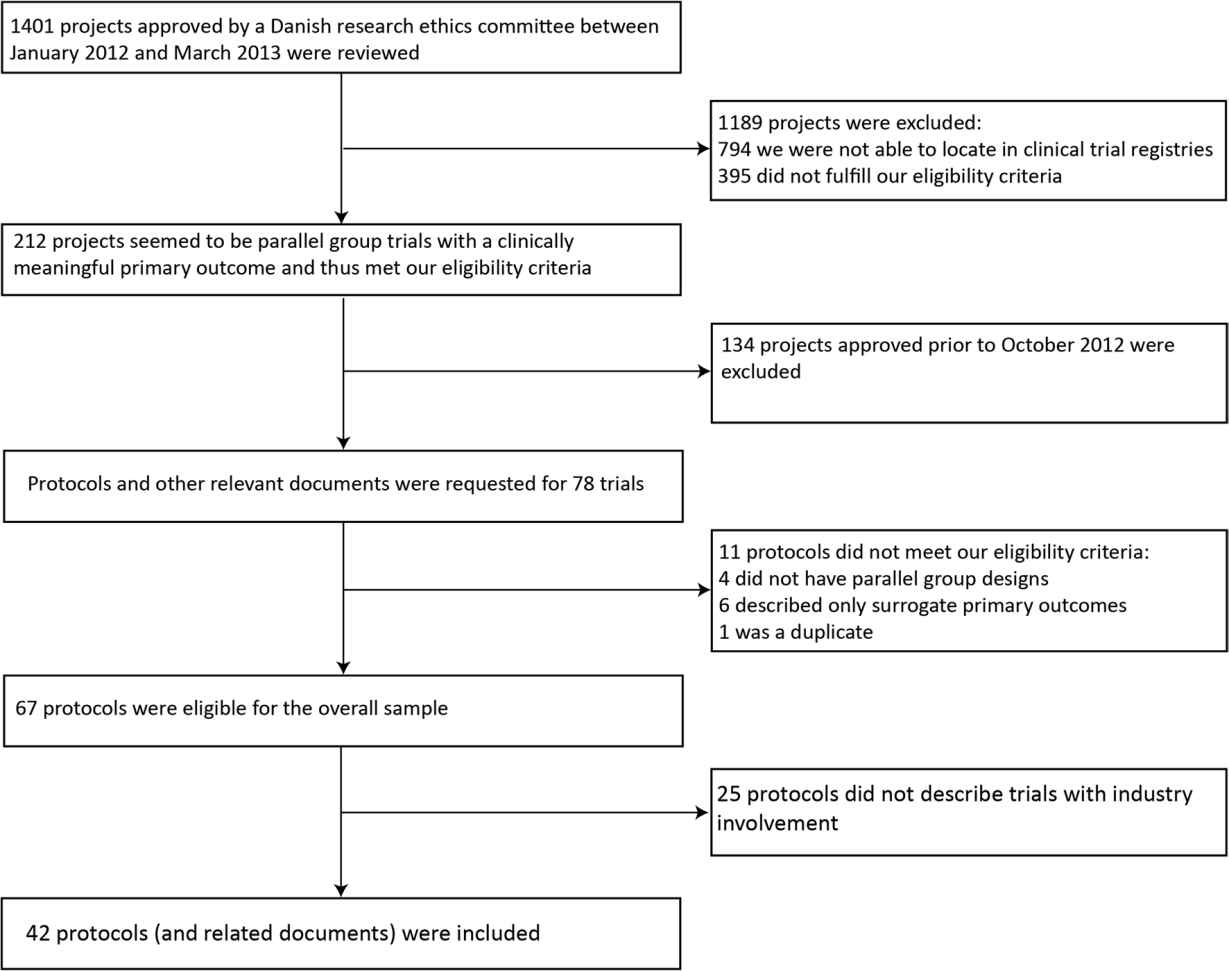
Flowchart of included protocols

We identified 1401 trials approved by ethics committees in Denmark between January 2012 and March 2013. Of those, we excluded 1,189 trials because we were not able to identify them in trial registries (n = 794) or because they did not meet our eligibility criteria (n = 395). The remaining 212 trials appeared eligible, but we could not realistically extract data from so many trials, so we limited the timeframe to October 2012 to March 2013. The resulting sample was 78 trials for which we applied for access to clinical study protocols and other documents through a Freedom of Information request. Of these, we excluded 36 trials; 10 because they did not meet our eligibility criteria; one because it was a duplicate; and 25 because they did not have any industry involvement. Thus, our final sample was 42 trials. The process is summarised in Fig. 1.

### Characteristics of included trials

Thirty-nine of the 42 included trials (93%) were multi-centre trials. The median planned sample size was 576 participants (IQR: 361–1130 participants). Twenty-nine of the included trials (69%) were drug trials, 6 (14%) tested devices, one (3%) a type of surgery, and six (14%) were classified as ‘other’. Trial characteristics for partially and fully industry sponsored trials are shown in Table 1.

**Table 1:** Characteristics of included trials

	Total (n = 42)	Partially industry sponsored (n = 10)	Fully industry sponsored (n = 32)
Type of trial			
Multi-centre	39 trials (93%)	9 trials (90%)	30 trials (94%)
Single centre	3 trials (7%)	1 trial (10%)	2 trials (6%)
Planned sample size			
Median	576 participants	275 participants	641 participants
Interquartile range	361–1130 participants	172–781 participants	407–1217 participants
Type of intervention examined			
Drug	29 trials (69%)	2 trials (20%)	27 trials (84%)
Device	6 trials (14%)	2 trials (20%)	4 trials (13%)
Surgery	1 trial (2%)	0 trials (0%)	1 trial (3%)
Other	6 trials (14%)	6 trials (60%)	0 trials (0%)

### Access to data and publication constraints

The roles and responsibilities of the sponsor and investigators were described in some detail in 20 of 42 trials (48%). Thus, for more than half of trials (n = 22, 52%) it was not clear which role the sponsor had in the project, apart from providing the funding.

### Accumulation of data and early stopping

In 8 trials (19%) we were certain that the sponsor had the opportunity to review data during the study and in 14 trials (33%) we were confident it was not possible. In the remaining 20 trials (48%) it was unclear. In 27 trials (64%) it was mentioned that the sponsor could stop the trial early and in 15 trials (36%) early stopping was not mentioned in any of the documents. In 23 of the trials (55%) the sponsor could stop the trial for any reason, in two trials (5%) specific reasons for stopping were mentioned, and in two trials (5%) it was unclear whether specific reasons were needed. The specific reasons mentioned were, for example, “reasonable medical or administrative reasons”, “futility” and “benefit”.

### Ownership of data and rights to publish

In 20 trials (48%) it was clear that the sponsor owned all data accumulated during the trial and in six trials (14%) the investigators owned the data. In the remaining 16 trials (38%) it was unclear who owned the data. Of the 32 fully industry sponsored trials, the sponsor owned the data in 19 trials (59%).For the remaining 13 trials (41%), ownership of data was unclear, whereas the investigator did not have ownership of data for any of the fully industry sponsored trials. Ownership of data was not mentioned in the ICDs for any of the included trials.

Investigators’ right to publish was explicitly constrained in 30 trials (71%), explicitly unconstrained in 7 trials (17%), and unclear in the remaining 5 trials (12%). In fully-industry sponsored trials there were explicit publication constraints for 29 out of 32 trials (91%) while for partially-industry sponsored trials only 1 of 10 (10%) had explicit publication constraints. The constraints on publication rights were not mentioned in the ICDs for any of the 30 trials with publication constraints (Table 2). The types of publication constraints are described in Table 3.

**Table 2:** Results from included trials

	Total (n = 42)	Partially industry sponsored (n = 10)	Fully industry sponsored (n = 32)
Roles and responsibilities of sponsor described			
Yes	20 trials (48%)	6 trials (60%)	45 trials (44%)
No	22 trials (52%)	4 trials (40%)	18 trials (56%)
Owner of data accumulated during the trial			
Sponsor	20 trials (48%)	1 trial (10%)	19 trials (59%)
Investigator	6 trials (14%)	6 trials (60%)	0 trials (0%)
Unclear	16 trials (38%)	3 trials (30%)	13 trials (41%)
Sponsor had the opportunity to review data during trial			
Yes	8 trials (19%)	0 trials (0%)	8 trials (25%)
No	14 trials (33%)	7 trials (70%)	7 trials (22%)
Unclear	20 trials (48%)	3 trials (30%)	17 trials (53%)
Sponsor had the opportunity to stop the trial early			
Yes, for any reason	23 trials (55%)	0 trials (0%)	23 trials (72%)
Yes, but only for specific reasons	4 trials (9%)	0 trials (0%)	4 trials (13%)
No	7 trials (17%)	6 trials (60%)	1 trial (3%)
Unclear	8 trials (19%)	4 trials (40%)	4 trials (12%)
Rights to publish were constricted			
Yes	30 trials (71%)	1 trial (10%)	29 trials (91%)
No	7 trials (17%)	7 trials (70%)	0 trials (0%)
Unclear	5 trials (12%)	2 trials (20%)	3 trials (9%)
Publication constraints mentioned in ICDs (n = 30)			
Yes	0/30 trials (0%)	0/1 trial (0%)	0/29 trials (0%)
No	30/30 trials (100%)	1/1 trial (100%)	29/29 trials (100%)

**Table 3:** Types of publication constraints described for included trials

Type of publication constraints	N = 42
Publication not allowed for a pre-specified time period	22 trials (52%)
Sponsor can review potential publications or presentations	30 trials (71%)
Sponsor can comment, but investigators must not comply with comments	14 trials (33%)
Sponsor can comment, and investigators must comply with comments	13 trials (31%)
Sponsor can delay publication	21 trials (50%)

All results can be seen for partially and fully industry sponsored trials, respectively, in Table 2.

## Discussion

We found that in almost all fully industry sponsored trials (91%) in our sample, the investigators’ right to publish was explicitly constrained in some way. The most common types of constraints were that the sponsor had the right to review potential publications; that investigators could not publish results for a period of time; and that the sponsor could delay potential publication. In one third (31%) of the included trials, the sponsor could comment on potential publications and the investigators needed to comply with the comments. In all fully industry-sponsored trials where determination of data ownership was possible, the sponsor explicitly owned the data. In none of the included trials were ownership of data or publication constraints mentioned in the ICDs.

We also found that in 19% of trials the sponsor could review data during the trial, and as the sponsor could stop the trial for any reason in 55% of trials, this meant that the sponsor had the opportunity to stop the trial based on interim results and potentially without using pre-defined criteria. Trials stopped early might be misleading, e.g., in 2011, Eli Lilly voluntarily withdrew drotrecogin alfa from the US market. The drug was approved based on a trial that was stopped early due to apparent benefit. However, a subsequent post-marketing trial found no significant benefit [17].

### Relation to previous research

In 2006, Gøtzsche et al. showed that out of 44 industry-initiated trials approved in Denmark in 2004, 41 (93%) had publication constraints. Similarly, Kasenda et al. have shown that out of 456 protocols approved by ethics committees in Switzerland, Germany, and Canada between January 2000 and November 2003, 393 (86%) described an industry partner’s right to disapprove or review the manuscript. Our study replicates these findings in a recent sample of trials. Additionally, to the best of our knowledge, this study is the first study to examine whether publication constraints are communicated to research participants, which was never the case.

### Limitations

Our study has important limitations. First, for a relatively high number of trials we did not have sufficient information to assess all criteria, e.g. it was unclear whether the sponsor could accumulate data in 48% of included trials. Additionally, some of the assessments were subjective and while all assessments have been checked by a second author and we tried to be conservative, this should be taken into consideration. Second, as the website of the National Committee on Health Research Ethics only contained relatively limited information, we were not able to identify a substantial amount of potentially eligible trials described on the web site when we searched for them in clinical trial registries and if these trials were systematically different from the trials we could identify, this might introduce bias in our sample.

Thirdly, while our sample is more recent than those used in other similar studies, more than eight years have passed since the trials were given ethical approval and standards for core documents to be evaluated by ethics committees for a clinical trial application might have changed. Likewise, it is possible that an increased focus on access to data and publication of results might mean that our results are not representative for trials conducted today, although there seem to have been little improvement from what has been found in previous studies compared to ours. All included trials were approved by ethics committees in Denmark, which might also limit the generalisability of our results, although almost all trials were multi-centre, multi-national studies. We are not aware of any reason that trials approved in Denmark should be systematically different from trial approved elsewhere.

Lastly, we had to sign confidentiality agreements to obtain access to CSPs and related documents, which means we are not able to share our more detailed data or provide in depth examples. This limits the transparency and reproducibility of our study.

### Implications for future research

As the process of getting access to protocols and extracting the data was very time-consuming, our sample is now somewhat dated; therefore, it would be relevant to examine to which extent the problems we identified are still present in trials conducted today. Nonetheless, Our study has several implications for research practice. As publication constraints seem to be widespread, the research community must consider whether this is an acceptable practice. Dissemination bias has been documented to be a widespread problem and publication constraints can contribute to this [3]. Additionally, early stopping of trials when the sponsor has access to data can lead to overestimation of the benefits. Ethics committees should ensure that interim analyses and DMCs are independent of industry sponsors. Finally, research participants should be fully informed about key aspects of trials, including data ownership and publication constraints in informed consent documents. As one of the primary motivations for participating in research is altruism [9, 10], this is important information that is necessary for true informed consent. However, while informing research participants of publication constraints is important, we believe that the primary solution to the problem should be to ensure that constraints on publication is no longer allowed in research that involves volunteers.

## Conclusions

Publication constraints are common in industry sponsored trials, and data from such trials is almost always specified to be owned by the sponsor. Additionally, the sponsor can often stop the trial for any reason and can sometimes review unblinded data while the trial is ongoing, even when explicit pre-defined stopping rules were not mentioned. The restrictions on publications imposed and rights to the data were not communicated to potential trial participants.

## Data Availability

The datasets generated and/or analysed during the current study are not publicly available as we needed to sign confidentiality statements in order to obtain access to the documents.

## Abbreviation

RCT: Randomised controlled trial
